# Complete Resolution of Granulomatous Rosacea With Topical Ruxolitinib

**DOI:** 10.7759/cureus.82457

**Published:** 2025-04-17

**Authors:** Jeffrey D Weiner, Sara Khoshniyati, Olivia Pierog, Sima Rozati

**Affiliations:** 1 Department of Dermatology, Johns Hopkins University School of Medicine, Baltimore, USA

**Keywords:** granulomatous rosacea, inflammatory skin disease, refractory rosacea, topical jak inhibitor, topical ruxolitinib

## Abstract

Granulomatous rosacea (GR) is a rare and challenging subtype of rosacea that resists conventional therapies. Here, we present the case of a 56-year-old woman with a two-decade history of treatment-refractory GR who experienced significant clinical improvement with topical ruxolitinib, a Janus kinase (JAK) inhibitor, formulated as a 1.5% cream applied twice daily. After failing multiple established treatments, the patient achieved near-complete resolution of her condition following 10 months of combination therapy with topical ruxolitinib and metronidazole cream. This case highlights the potential efficacy of topical JAK inhibitors as a novel therapeutic approach for recalcitrant GR.

## Introduction

Granulomatous rosacea (GR) is an uncommon variant of rosacea, characterized by firm facial papules and nodules accompanied by histopathological evidence of granulomatous inflammation [1]. Unlike typical rosacea, GR often follows a chronic, progressive course and responds poorly to conventional treatment modalities [2-4]. Its pathophysiology remains incompletely understood, though immune dysregulation and aberrant inflammatory pathways are believed to play central roles in disease development [1]. 

The management of GR poses a significant therapeutic challenge, with many patients experiencing persistent symptoms despite multiple treatment attempts [2-4]. Therapies include topical agents such as metronidazole, azelaic acid, and calcineurin inhibitors, as well as oral medications including tetracycline-class antibiotics, oral isotretinoin, and anti-parasitic agents [1]. However, treatment failure and recurrence are common, highlighting the need for novel therapeutic approaches for this recalcitrant condition [2-4]. 

Janus kinase (JAK) inhibitors have emerged as promising agents for various inflammatory dermatoses due to their ability to modulate multiple cytokine pathways involved in immune-mediated inflammation [5,6]. Although their efficacy has been established in other inflammatory skin conditions and rosacea subtypes, evidence supporting their use in GR remains limited [4-9]. This case report aims to contribute to the limited literature by documenting the successful treatment of long-standing, treatment-refractory GR with topical ruxolitinib, a JAK inhibitor formulated for topical use.

## Case presentation

We present the case of a 56-year-old Black woman with a two-decade history of GR and no other significant past medical history. 

The patient initially presented to our clinic at age 36 with a three-week history of a pruritic facial rash. Physical examination revealed tender, erythematous papules and nodules along the medial forehead, cheeks, chin, and perioral region. 

Multiple skin biopsies performed over several years revealed a perivascular and periadnexal lymphohistiocytic infiltrate. Compact, non-caseating granulomas with multinucleated giant cells and surrounding lymphocytes were observed in the mid-to-deep dermis, extending into the subcutis. These pathologic findings, in conjunction with the patient’s clinical presentation of persistent, erythematous papules and nodules on the face, confirmed the diagnosis of GR. Laboratory evaluations throughout the disease course were unremarkable with regard to the possible etiology of her skin condition. 

The patient’s condition was refractory to multiple standard rosacea therapies, including topical azelaic acid, tacrolimus, and ivermectin, as well as oral ivermectin, minocycline, and doxycycline. Several courses of isotretinoin led to transient improvement, but the patient self-discontinued multiple times due to worsening facial erythema and swelling, as well as joint pain. 

After two decades of unsuccessful treatment, the patient was initiated on a combination regimen of topical ruxolitinib 1.5% cream, applied twice daily, and topical metronidazole cream. Within six weeks, her skin showed marked improvement. After 10 months of treatment, she achieved near-complete resolution of her GR. 

The patient was closely monitored for potential adverse effects of topical JAK inhibitor therapy, including common application site reactions or folliculitis; no side effects were observed. There was no evidence of disease exacerbation after one year of follow-up, suggesting the durable efficacy of this treatment approach. 

**Figure 1:**
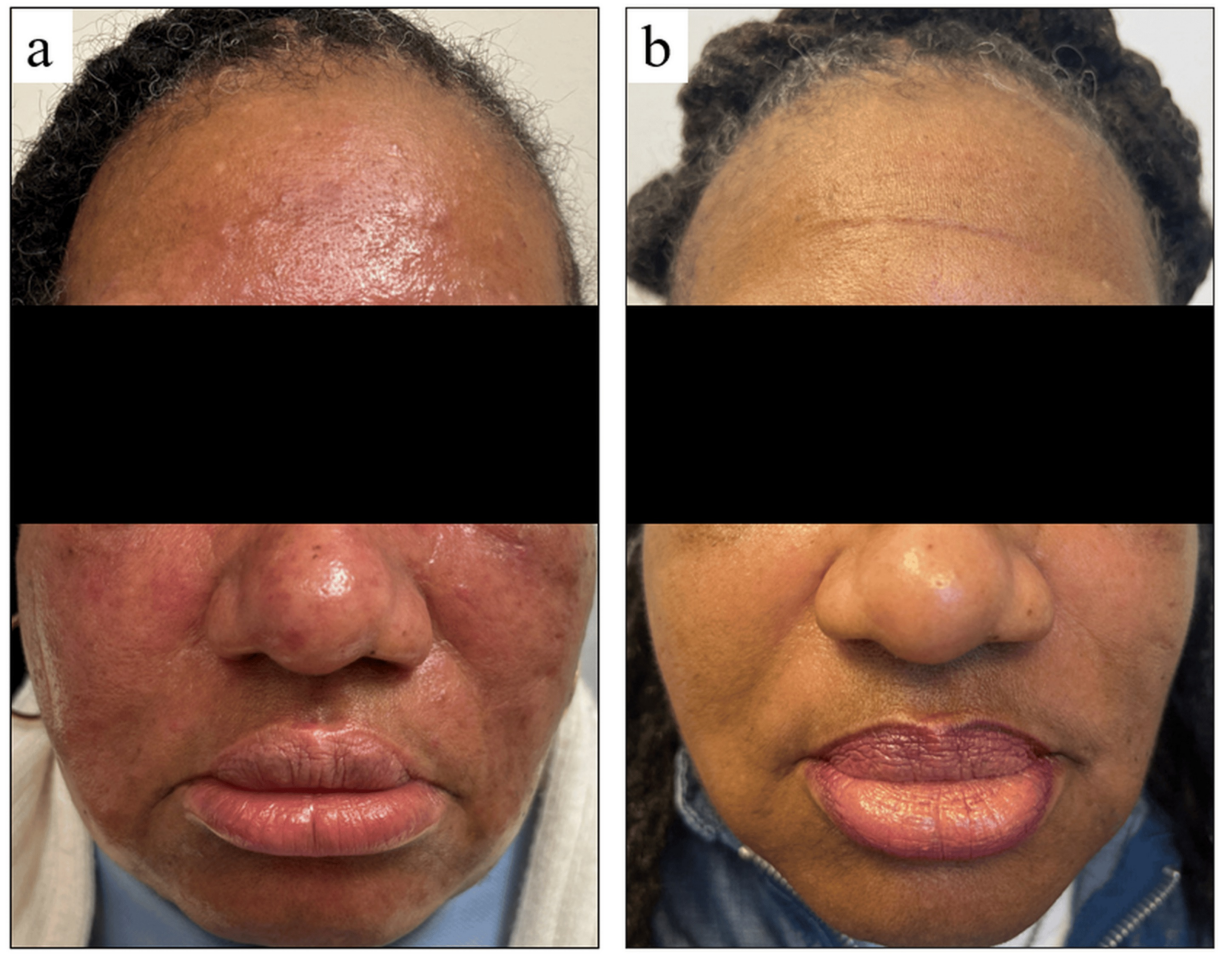
Facial photographs before and after treatment This figure shows the clinical response of the patient to topical ruxolitinib therapy. (a) Baseline image showing significant inflammation, erythema, and papules, taken after two decades of treatment failure and prior to initiation of topical ruxolitinib. (b) Image after 10 months of treatment with topical ruxolitinib, demonstrating near-complete resolution of granulomatous rosacea.

Clinical photographs captured before and after the treatment illustrate the significant impact of topical ruxolitinib on the patient's condition. The baseline image (Figure 1a) shows extensive erythematous papules and nodules, predominantly affecting the cheeks and chin, with associated swelling. The lesions were firm on palpation and exhibited pronounced granulomatous features. In contrast, the image taken after 10 months of treatment (Figure 1b) demonstrates a near-complete resolution of inflammation, erythema, and papules, with no palpable nodules.

## Discussion

GR is among the most challenging subtypes of rosacea to manage effectively [1]. Its chronic, relapsing course and poor response to conventional therapies contribute to significant distress and reduced quality of life. Our case illustrates the potential efficacy of topical JAK inhibitors, specifically ruxolitinib, in the management of treatment-refractory GR. 

JAK inhibitors exert their therapeutic effect by blocking the JAK-STAT signaling pathway, which mediates the activity of multiple proinflammatory cytokines implicated in the pathogenesis of inflammatory skin disorders [5]. Molecular studies have further implicated STAT1, STAT2, and STAT3 activation in the pathogenesis of rosacea [10-12]. By inhibiting this pathway, JAK inhibitors attenuate this inflammatory cascade. 

The literature on JAK inhibitors for rosacea has been evolving, with several studies investigating systemic oral JAK inhibitors such as abrocitinib, upadacitinib, and tofacitinib in the general management of rosacea [4,7-9]. However, evidence specifically supporting the use of topical JAK inhibitors for granulomatous rosacea remains extremely limited. One prior case report has documented successful treatment of GR with systemic oral abrocitinib, showing substantial improvement after 20 weeks of therapy in a 53-year-old woman with persistent facial erythema and burning sensations, without recurrence or adverse effects during follow-up [4]. Additionally, topical tofacitinib has shown efficacy for rosacea in a mouse model, with a significant reduction in erythema, inflammatory cell infiltration, and dermal angiogenesis [9]. 

Topical JAK inhibitors offer potential advantages over oral formulations, including a more targeted delivery directly to affected skin areas while minimizing systemic absorption [13]. This localized action is expected to reduce the risk of serious systemic adverse effects, such as thromboembolic events, infections, and laboratory abnormalities that have been associated with oral JAK inhibitors [13,14].

To our knowledge, this is the first reported case of topical JAK inhibitor use in a human patient with GR. The rapid and sustained clinical improvement observed in our patient, who had previously failed multiple established therapies over two decades, highlights the potential of topical JAK inhibitors to address an important unmet need in the management of recalcitrant GR. Further research involving larger patient cohorts is warranted to confirm the efficacy and safety of topical JAK inhibitors for GR, as the current body of evidence is limited. 

## Conclusions

This case demonstrates the efficacy of topical ruxolitinib as a potential therapy for refractory GR. Our patient achieved near-complete resolution of long-standing symptoms after failing multiple conventional therapies over two decades. While further research is needed to validate these findings, topical JAK inhibitors may offer a promising therapeutic option for patients with recalcitrant GR. This case contributes to the emerging evidence supporting the role of JAK-STAT pathway modulation in inflammatory dermatoses and highlights the need for continued investigation into targeted therapies for challenging rosacea subtypes.
